# The Relation of the Profession to the Secular Press and the Rostrum

**Published:** 1874-11-01

**Authors:** Thos. D. Washburn

**Affiliations:** Hillsborough, Ill.


THE
### MEDICAL EXAMINER.
A
Semi-Monthly Journal of Medical Sciences.

EDITED BY N. S. DAVIS, M.D., AND F. H. DAVIS, M.D.

No. XXI. CHICAGO, NOV. 1, 1874. VOL. XV.

### Original Communications.

### THE RELATION OF THE PROFESSION TO THE SECULAR PRESS AND THE ROSTRUM.

AN ADDRESS READ BEFORE THE CENTRAL ILLINOIS MEDICAL ASSOCIATION, BY THOS. D. WASHBURN, M. D., HILLSBOROUGH, ILL.

OUR worthy President has assigned me the duty of preparing a paper on "The relation of the profession to the secular press and the rostrum;" a topic involving very little anatomy, though I propose to unearth a skeleton, and as I proceed to discuss the pathology, which naturally antedates most skeletons, I may attempt to apply some therapeutics, which involves more or less a knowledge of physiology.

I am conscious that I have a delicate task, for it is a question only recently sprung, and is liable to much misconstruction. To many minds a new idea is as dangerous as nitroglycerine, and any reconstruction or meddling with well-established usages is as perilous as the restored pillory in "My Novel," of Bulwer Lytton. But, gentlemen, the men who do not think and cannot see how the past can be improved, and believe that progress is an enemy, unworthy our confidence or examination, are not fit to practice medicine, and should make no pretensions to science. So far as the various branches which make up the science of medicine are concerned, you readily admit an immense progress, you seek for the highest culture, you consult the most advanced scholarship, the best authorities. But when you are asked to modify a rule of action, a custom of half a century; when the experience of two generations presses upon you, and the altered circumstances of all your surroundings invite a change; when the social, political, and religious life has assumed a dozen different phases, adapting itself to the peculiar forces and national condition and demands of the times, does it become us to doze on in our Rip Van Winkle sleep and ignore the light of the nineteenth century? It is astonishing with what ease we wrap the mantle of self-complacency around us, and fondly believe that we are the only true medical light of the world; that medical wisdom cannot be generated outside of the regular profession; that it is conceived, gestated, and brought forth only in the legitimate medical fold. Why, the greatest advances we have made, the most famous epochs in our history, as a profession, have been when some Parthian arrow has been shot from an enemy, when some grand broadside has been given us from a school of quacks or band of charlatans.

That despised, ignorant, and pugnacious Thompson, years ago, with a single idea and formula, untenable at that, viz.: that "Heat is life, cold is death," threw a bomb-shell into our camp that unhinged and disjointed the whole theory and practice of the day; sent calomel and the lancet to the rear, and changed the whole medical front. An exclusive botanic practice arose on the debris, and lives in varied forms to-day, eclecticism being one of its most promising children. It largely modified regular practice, and we are almost unconscious how much good we owe to its venom and acrimony; this blustering storm, which came down so suddenly on our old craft, made us throw overboard some antiquated notions and old rubbish, retrim the ship, mend the sails, and study more carefully the winds and currents in which we were moving. Hardly a score of years elapsed, before another medical formula struck us like an iceberg in a summer fog, "Similia similibus," a more monstrous absurdity than the other, but it taught us, oh, how much! Many who hear me to-day will testify that in their pupilage they were taught to believe disease to be an entity, and that *medication* alone could exorcise it. We never thought of asking how this or that disease would result if let alone; it was the medicine that restored and saved. True we had some vague notions about the *vis medicatrix naturæ*, but, as a whole, nature got little credit for her labor; associated with this "*similia*," came the *infinitesimal;* that was a revelation; logic, data, philosophy could not reach it; common sense could bang its brains out and fail to strike it; it was beyond the reach of analysis; chemistry it had none; its particles were so comminuted that nothing but the eye of imagination could appreciate them.

We could make some stagger at *mesmerism;* the snapping doctor *did* something, he put the air in motion; we could conceive of a *faith* cure; even the *royal* touch for scrofula could be guessed at, but an *infinitesimal* was too much for us. The vulgar mind could realise a sensible effect in lobelia or cayenne; but *similia similibus* in infinitesimal doses, with all its foreign pretension, vast erudition and decillionth accuracy, captivated the wise, the great and good; its air of mysticism, its beautiful attenuation, its mysterious potency, baffled the power of all ordinary ratiocination, and thousands were overpowered by its imposing claims and vast nothingness. But it taught the profession that very much we had attributed to medication was not entitled to any such agency; that the largest number of diseases were self-limited, and little or no medication was better than much.

Among the elements of varied success which have developed and maintained these two schools has been the secular press. Both appealed to the prejudices and weakness or ignorance of the popular mind; the one putting up a man of straw, mineral medicine and the bloody lancet, the other the harmlessness and ease of its administration. So far as my observation extends, no editor appeals to reason, philosophy, or common sense in support of either.

No challenge is ever made through the press to honestly discuss the merits of either. Why then is the secular press partial to these *false* systems? For the obvious reason, they fill their pockets. The advertisements of irregular medicine, its puffs and locals are among the principal sources of wealth which sustain them. A man is not going to abuse another who feeds his children, and puts silk dresses on his wife, and supplies him with fast horses. The most reputable and disreputable papers in the city, as well as rural districts, teem with this class of advertisements, the religious press not excepted. These quack kings, Jaynes, Townshend, Radway, Leonidas Hamilton, even Pierer, of Buffalo, and scores of others, spread their money like water; a thousand lesser lights rush in, steal the name of some distinguished Professor, or claim some grand discovery, authenticate the same with cabinet officers and D.D.'s, and keep the public on the *qui vive* for the next astounding medical sensation. They appeal to man's lower and higher nature; no local, religious, or social prejudice is left forgotten; you have Temperance *Bitters* by Walker, Quaker Bitters by Flint, and Plantation Bitters by *Skinflint;* there is no field where successful lying reaps richer harvests. Then you have the *traveling* medical mountebank, with his posters and puffs, stuffing the pockets of the press and skinning the poor invalid. Your *Wizard Oil* and other medical delusions move on with the blare of trumpets and the pageantry of fine equipages. Your more aristocratic and imposing Water Cure, Health Lift, Russian Bath, and semi-heretical delusions and institutions spread their wonders and triumphs broadcast over the land, through the press; surely you cannot expect them to break up their own feed troughs!

But let us look into the philosophy of this matter. Says a distinguished divine, "I now declare that I consider the newspaper to be the grand agency by which the gospel is to be preached, ignorance cast out, oppression dethroned, crime extirpated, the world raised, heaven rejoiced and God glorified." If such are the views of the Rev. Talmage in regard to the influence of the press on the moral and religious character of the nation and the world, how much more appropriate when applied to the *medical* sentiment, which is more exclusively educated by the false teachings of the press!

There is no denying that the press is an immense power in the land; its influence on the public mind cannot be estimated; it enters the palace and hovel alike; the little child as well as the aged sire drink at its fountain; baited with the minutest portion of truth to cover the ugly hook of medical error, the insinuating style of these pretenders is bound to warp the reason and taste of the public; false doctrine so constantly reiterated, with all the blandishments of culture and genius, will deceive the very elect.

(Distinguished names have been blotted from our roll of honor by the brilliant success which charlatanry occasionally achieves.)

The school boy gets the annual almanac in twenty varied forms; fun and anecdote on one side, marvelous cures and marvelous medicines on the other; the farmer runs over his weekly with the aches and pains of protracted labor still on him, and listens to the syren song of rheumatism cured in twenty minutes; neuralgia annihilated in forty seconds, "to be found at any drug store." The merchant at the close of business, or after his comfortable dinner, seizes his *daily* and next the markets he finds Dr. Pelham's cure for sick headache, colic, gravel, and liver complaint, with the most imposing testimonials, and so on *ad infinitum.* Will any one tell me that the public are not consciously and unconsciously educated to a variety of false medical belief? These thousand and one avenues which open from early dawn to dewy eve in every daily, weekly, and not a few of our monthlies, are sweeping away the grand old landmarks and filling their places with doubt, error and fanaticism. The rostrum fortunately is safe from much mischief in this direction. There is no law forbidding us to let our light shine on the *rostrum*, and where no law is there is no sin. Science and reason are too large an element on the rostrum for it to be often prostituted to base pretension and imposture; the district lyceum and the popular lecturer must minister to our good sense or to empty seats; sophistry and delusion here can be throttled before they get to be respectable snakes; not so with the newspaper: it comes with the breath of the scourge and carbolic acid won't neutralize it.

I have spoken of the press with its swollen stream of contaminated and worthless medical literature, as exhibited in the daily and weekly throughout our land. One word as to the quacks and ignoramuses who practice in almost every hamlet and afflict every community; as a class they excel in electioneering; they are always before the people; seldom before their books; they know every man, woman and child that crosses their pathway. Talk about *blarney:* Pat and Biddy are at a discount; they are eminently road sweepers; they know every man's farm and circumstances, his nativity, religion and politics, when and to whom married, his horses, mules and cattle, can call by name his very dogs; constant contact with men gives them much apparent ability and they have an influence which may well be respected; cordial, caressing, conservative, seldom radical even in medicine, never positive and declaratory except they know well the individual or the crowd, they often become an oracle to the credulous and simple.

Such is the element we as a profession have to meet. Their influence is as pervading as the miasm of an Indian jungle. The frothy gossip which exhales from these medical vampires is as pestilent and contagious as the cholera or yellow fever. They are conspicuous; like small tradesmen they show all they have and exhibit their wares to the best advantage. They are perennial, the regulars occasional; they are persistent, the regulars spasmodic; they blow a steady breeze, the regulars gusts; they use *common* names and try to make themselves understood by simple illustrations; they call stomach *stomach*, not the gastric viscus; salt *salt*, not chloride of sodium. Their intimate relation to the masses, their appeals to prejudice, their misrepresentation of facts, produce results and warp the judgment of many honest minds. What is the remedy? Shall they be fought with their own weapons? Partially, yes; largely, no; what recourse have we? The attainments and general culture which are conceded us should not be so very modestly concealed as our ethics seem to imply and many of our members so rigidly observe; our personal and general influence should be more sensibly felt on all the medical questions of the day; but of all our resources none is more legitimate than the proper use of the secular press. What is the press? The reflection of the best thought, the practical wisdom, the grand results of the age. The ablest statesmen and divines, the philosophers and most advanced scientists, and the highest business interests of the land and the age cluster round the press. The world seeks light; the American mind, active, dashing, pushing, reports, interviews, telegraphs, and turns creation upside down for news.

Next to air, water and sunlight, the newspaper is essential to the existence of our people. Government, commerce, education, religion, science, agriculture, mechanics, art, and every industry of the land, breathe, live and glow in the secular press. But we, a fraction of humanity, represented by sixty-one colleges, seventy-two journals, some twenty thousand practitioners, and twenty millions or more of patrons, dare not lisp a syllable outside of *hygiene*, lest some venerable *Gusticutus* or sharp Rusticus pounce on our temerity, cry halt! and threaten us with instant and eternal exclusion from all that is reputable, regular and infallible in medicine. It does seem to me about time to put off our swaddling clothes and put on the habiliments of men. This never going into water until you have learned to swim may be good advice to children but it is hardly the stuff for grown people.

The thirty-nine articles doubtless were good when they were born, but do n't you think the *nine* could be dropped to advantage?

Do n't you believe the Westminster Catechism could be slightly altered and not shock the Christian intelligence of the day? No one excels me in true reverence for the past. I am no revolutionist, anarchist, or idle agitator, but I think the time has come to forsake false gods. Those who choose to worship medieval fancies and blindly bow the knee to moss-covered Diana's, should have the privilege, whether social, political, medical or religious; but with all the light of the present age it seems possible that even the medical ethics, habits and precedents of the past generation might be modified, and possibly better adapted to the wants and necessities of the hour. I may be mistaken. I have ceased to worship *age* for its intrinsic excellence; *distance* never presents those rose-colored hues that make vice virtue, or deformity perfection. I am not for mixing homœopathy, eclecticism, or any other absurd and visionary dogma with regular practice, but I am in favor of informing the public what regular medicine is, and giving them a better opportunity to judge correctly of its merits, to separate the wheat from the chaff and stand out more boldly in defence of our doctrines and our rights; to challenge discussion on the merits of our position; to make ourselves aggressive as well as defensive; to shape and mould and vitalize public medical opinion rather than see it submerged by error and fraud.

It is proper and right that custom and usage should be formulated, and general principles laid down for corporate action and ordinary emergencies; but in this age of development, change and progress, we cannot expect any formula to outlive its usefulness; after the chicken is hatched what is the use of the shell? We concede that we have learned much from our opponents; they have indirectly been the cause of great advancement; we are actually better for their criticism and censure; but they hold that remedies are *personal* property, and any combination entitles them to secresy, private use and a *patent*, which must not be infringed; we hold the opposite, that every remedy is *public* property, and boldly publish the same to the world. They seize the very article we have announced, trump up a fancy name, and impose it on a credulous public as a wonder, a panacea, a life restorer, and with letters-patent or otherwise, reap vast sums of money from their ready dupes. Which course is the more humane? Which course should the public approve? Which course should a discriminating, honorable and appreciative press sanction?

An ignoble deception is sent broadcast over the land, through the press, for pay. That is the motive power which afflicts the secular press. The knaves reap a rich harvest and divide with the press. The people are misled by the press and the whole practice of medicine brought into disgrace. We cannot afford to pay for chasing up these false statements and impositions, consequently our corrections are declined. A *v* or an *x* will open their columns to the next impostor, and so the public are educated and a premium placed on deception, fraud and ignorance, and a low, false, cheap system of practice begotten by this popular mis-education.

We have men abundantly qualified to announce these facts and show up the true position of the profession, either in our dailies, weeklies or monthlies, but the precedent that the true physician should not publish anything in regard to his calling, except in the legitimate medical journal, has existed so long that it would be rash and perilous for a reputable M.D. to attempt to contend for his rights, or define his position, in the secular press or *monthly*.

Our code of ethics is as faultless as any document of its age; it is largely what the profession need, but somehow false views, and inferences, and practices, have been drawn from it; we shall soon be, if not already, in the dilemma of some of our good presbyterian brethren, having a *formula* but differing essentially in *reality;* it certainly is eminently proper that *each* generation should leave its impress on it, lest they be misunderstood. I

presume it has not escaped your notice that there has been a certain restlessness about this matter existing among prominent members of the profession, and for a year or more agitating the journals, even the staid and respectable *Boston Medical and Surgical Journal* has been somewhat exercised, the New York and Philadelphia journals receiving some gentle reprimands for their fast proclivities; quite a variance has been manifested as to the *amount* of popular medical instruction the people should receive; some were for homœopathic, others heroic doses of this pabulum. My own opinion is that our ethics are misconstrued; that they give more latitude than most. of the profession have been inclined to take. It says, under "Duties of the profession to the public," art. 1st, paragraph 1, "As good citizens, it is the duty of physicians to be ever vigilant for the *welfare* of the community and bear their part in sustaining its institutions (newspapers) and burdens."

"They should also be ready to give counsel to the public in relation to matters especially appertaining to their profession, as on subjects of medical police, public hygiene, &c., —— in regard to measures for the prevention of epidemic or contagious disease." (4) "It is the duty of physicians who are frequent witnesses of the enormities committed by quackery, and the injury to health, and even destruction of life, caused by the use of quack medicines, to enlighten the public on these subjects, to expose the injuries sustained by the unwary from the devices and pretensions of artful empirics and impostors."

If here is not a *carte blanche* for any attack we may choose to make through the press, rostrum, or otherwise, on the multitudinous forms of medical deviltry which afflict the community, then the English language is out of joint. As *good citizens* we are called upon to be "*vigilant for the welfare of the community.*" Are you vigilant when you let false views and false practice, and imposing medical rascality and pretension come in like a flood and undermine and subvert the truth? When you see men clinging to foolish and baseless dogmas, and trusting life itself to consummate ignorance and unskilled and reckless presumption? We are verily guilty in neglecting to sound the alarm and awaken public sentiment to the audacious practices and destructive forces which are operating on society for want of information and enlightenment; both in regard to diseased conditions and the proper means of cure.

The public ought to know on sight a quack as well as they know a blackleg or a preacher; how one can drop down in a community and remain twelve months and not be detected is a mystery to me; a man that uses his tongue or his pen should be found out by that time. One thing we do know, that where you find a healthy itinerant it is presumptive evidence that he is no earthly account, and unable to make an honest living at home.

To return to the press: the question remains, how can it best subserve the interests of legitimate medicine? Not certainly by confining ourselves to the medical journals and making them the medium of our efforts to reach the popular mind; not solely by improving ourselves and rendering the profession *worthy* of all confidence; not by inveighing against all species of quackery; (for they might charge us with being interested witnesses); not by abusing the press and denouncing them as selfish and indifferent to the public weal; but by cool, dispassionate logic, a simple presentation of facts in the utmost fairness, selecting well-chosen abuses, follies and false notions, seizing the vulnerable points of error and placing them in popular form before the people; giving instruction in hygiene, the abuse of remedies, and all the shades which quackery assumes, regular or irregular; for we cannot ignore the fact that the members of the profession too often give occasion to just criticism and reproach. The address of the late presiding officer of the N. H. State Medical Society was mainly aimed at these, and our own personal knowledge is not exempt from much that is reprehensible, unworthy, and wrong, in this direction: impressing patients with the idea that they are worse than they are, thus substituting fear and anxiety in place of hope and cheerfulness; depreciating and undermining a brother practitioner by statements and insinuations that have but a modicum of truth; an imposing array of successful cases and profuse assertions of wonderful ability; approaching men and invalids without invitation and *volunteering* advice or medicine; slipping up on the blind side of a man's political, religious, or sectional bias, and soliciting favor; all these are a shame and disgrace and proper subjects of newspaper criticism; but we have a still further mission to perform. It is our business to *create* a medical sentiment, to put the people in possession of right doctrine, to educate and develop, not only in hygiene and physiology, but give them general principles in practice and therapeutics. It certainly is better they should learn from *us* than by the ignorant or designing pretender and demagogue.

Dr. Logan, President of the National Medical Association, ('73) says the "only channels" by which the people can be reached are the "*newspaper* and lecture room;" "this is our work for the future, to educate the people." The President of the Ohio State Med. Society, the same year, advises the daily paper to employ an eminent medical writer to occupy a column, and expresses the opinion it would do more good in educating a proper medical sentiment among the people than all the medical journals combined.

In a paper read a year ago last June, before the Montgomery Co. Medical Society, I use this language: "We number ten to one of our enemies, but they have captured the press, and by their persistent noise and bluster, confuse the public and paralyze the truth. We have county, district and state organizations, but we are hedged in by such an oppressive sense of our dignity, such solicitude for our position and ethics, such exclusiveness for our professional rights and decorum, that we have not given *legitimate* publicity to much of our labor and practice, thereby depriving ourselves of public sympathy and confidence.

"We can better shape public *medical* sentiment than lawyers can the *political*, or clergymen the *theological*, for we number more and have better access to the masses, and it is from sheer neglect we have allowed such a false state of things to exist; we have *slept* while the enemy has sowed tares." Again, an editorial in the *Boston Medical and Surgical Journal*,

January, 1874, closes with these words: "Nothing is further from our wishes than that the profession should expose itself to defilement by contact with politics, but as guardians of the public health, as the best judges on many points of morality, physicians, as a class, have a right to a voice in many matters. If we claim this consistently, moderately but persistently, it cannot be denied us. If we do not claim it we do not use all the means at our command for the benefit of society and the honor of the profession, and are false to the duty we owe both." Let me ask in all candor, would not the same facts and the same logic lead *us* to use the secular press to communicate our views and give instruction to the people?

As societies can we contribute to this end? Certainly we can; each local society could have papers written for this special purpose. Topics could be selected and a committee of one, two or three, appointed to prepare matter and give such facts as would enlighten the public mind, not only on hygiene, but much that pertains to the profession; their relations to each other, irregulars, and the public. The same could be done by the district and state society, and the medical profession brought in closer harmony and sympathy with the people, and the best *local* and most popular dailies made the medium of such communications. Our best writers could be instructed to prepare more elaborate papers for the popular monthlies or reviews, and a safe, healthy, medical literature permeate the reading matter which has such ready access to all classes. If these views are utopian, pardon my temerity; if correct, accept and adopt them.

